# Docetaxel, cisplatin, and fluorouracil with pegfilgrastim on day 3 as neoadjuvant chemotherapy for esophageal cancer

**DOI:** 10.1002/cam4.6974

**Published:** 2024-02-01

**Authors:** Osamu Maeda, Satoshi Furune, Mitsuro Kanda, Kazushi Miyata, Dai Shimizu, Shizuki Sugita, Kazuki Nishida, Masahiko Ando, Yasuhiro Kodera, Yuichi Ando

**Affiliations:** ^1^ Department of Clinical Oncology and Chemotherapy Nagoya University Hospital Nagoya Japan; ^2^ Department of Gastroenterological Surgery Nagoya University Graduate School of Medicine Nagoya Japan; ^3^ Division of Surgical Oncology, Department of Surgery Nagoya University Graduate School of Medicine Nagoya Japan; ^4^ Department of Advanced Medicine Nagoya University Hospital Nagoya Japan

**Keywords:** 5‐fluorouracil, cisplatin, docetaxel, esophageal cancer, pegfilgrastim

## Abstract

**Purpose:**

A high risk of febrile neutropenia (FN) from neoadjuvant chemotherapy with docetaxel, cisplatin, and fluorouracil (DCF) for esophageal cancer has been reported. The optimal timing of prophylactic use of pegfilgrastim remains to be elucidated. To evaluate the effect of pegfilgrastim administered on day 3, we conducted a feasibility study.

**Methods:**

Chemotherapy consisted of intravenous administration of docetaxel (70 mg/m^2^ per day) and cisplatin (70 mg/m^2^ per day) on day 1 and continuous infusion of 5‐fluorouracil (750 mg/m^2^ per day) on days 1–5. Pegfilgrastim was given as a single subcutaneous injection at a dose of 3.6 mg on day 3 during each treatment course. This regimen was repeated every 3 weeks for up to a maximum of three courses. Prophylactic antibiotics were not needed but were allowed to be given at the discretion of the physician. The primary endpoint was the incidence of FN.

**Results:**

Twenty‐six patients were administered DCF in combination with pegfilgrastim on day 3. After the first course of DCF, 10 out of 26 patients (38.5%) experienced grade 4 neutropenia, and two patients (7.7%) experienced FN. Of the 14 patients who did not receive prophylactic antibiotics, four had grade 4 neutropenia, including two who developed FN. On the contrary, of the 12 patients who received prophylactic levofloxacin, six had grade 4 neutropenia, but no cases of FN were observed.

**Conclusion:**

Administration of pegfilgrastim on day 3 was not sufficient to prevent FN due to DCF treatment, and prophylactic administration of both pegfilgrastim and antibiotics could be a solution.

## Graphical abstract

Although neoadjuvant chemotherapy with docetaxel, cisplatin, and fluorouracil (DCF) for esophageal cancer has been proven to be effective, a high risk of febrile neutropenia (FN) has been reported. To evaluate the effect of administering pegfilgrastim on day 3, we conducted a feasibility study. The results showed that administration of pegfilgrastim on day 3 was not sufficient to prevent FN due to DCF; prophylactic antibiotics were also required.

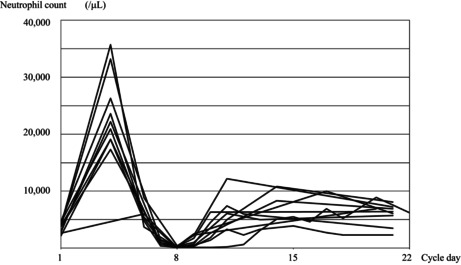

## 1 INTRODUCTION

In 2019, the global count of esophageal cancer cases reached 535,000, leading to 498,000 fatalities.^1^ The standard treatment in Japan for stage II/III squamous cell carcinoma (SCC) involves preoperative chemotherapy with 5‐fluorouracil (5‐FU) plus cisplatin (FP) followed by esophagectomy.^2^ A recent trial, JCOG1109 (NExT), demonstrated that administering neoadjuvant therapy with docetaxel, cisplatin, and fluorouracil (DCF) significantly enhanced overall survival compared to FP.^3^, ^4^

Nevertheless, DCF therapy poses a substantial risk of adverse events, such as neutropenia and febrile neutropenia (FN). To mitigate FN risk and ensure compliance with triplet regimens, pegfilgrastim, a long‐acting granulocyte colony‐stimulating factor (G‐CSF), should be given at least 24 h after completing myelosuppressive chemotherapy.^5^ Our previous report indicated that prophylactic pegfilgrastim administration on day 7, 24 h after 5‐FU infusion, inadequately reduced FN risk, with a 29.7% incidence.^6^ Conversely, Ishikawa et al. reported that pegfilgrastim administered on day 3 effectively decreases FN risk.^7^ Consequently, we conducted a feasibility study on DCF with prophylactic pegfilgrastim administered on day 3 as preoperative chemotherapy for esophageal cancer, using FN incidence as the primary endpoint to ascertain the reproducibility of previous findings.

## 2 PATIENTS AND METHODS

### 2.1 Eligibility criteria

To be considered for inclusion in this study, individuals needed to satisfy specific criteria. Patients were required to have a histological diagnosis of esophageal cancer, encompassing SCC, adenocarcinoma, adenosquamous carcinoma, or basal cell carcinoma. Additionally, eligible participants had to fall within the age range of 20–75 years, exhibit an ECOG performance status of 0 or 1, and possess normal organ function (neutrophil count ≥1500/μL, hemoglobin level ≥8.0 g/dL, platelet count ≥100,000/μL, aspartate transaminase (AST) and alanine transaminase (ALT) ≤100 IU/L, creatinine clearance (CCr) ≥50 mL/min, and left ventricular ejection fraction ≥50% with echocardiography). Furthermore, the inclusion criteria specified the requirement of having cStage IB, cStage II, cStage III, or borderline‐resectable disease according to the UICC‐TNM 8th edition. Alternatively, patients with T4 and tracheal infiltration at the cervical/cervical chest boundary, or those with cStage IV disease along with supraclavicular lymph node metastasis and no other distant metastasis, were also eligible. However, individuals were excluded if they had a tumor at the esophagogastric junction with the main lesion in the stomach instead of the esophagus, T4 invasion other than at the cervical/cervical chest boundary, or distant lymph node metastasis except for supraclavicular lymph nodes.

The study adhered to the guidelines outlined in the Clinical Trials Act (the Ministry of Health, Labor, and Welfare, Japan) and the Declaration of Helsinki. Approval for the trial was obtained from the Institutional Review Board of Nagoya University Hospital (approval no. 2019‐0518). Before participating in the study, all the subjects provided written informed consent. The trial details were registered with the Japan Registry of Clinical Trials (jRCTs041190129).

### 2.2 Treatment

Patients underwent neoadjuvant chemotherapy involving docetaxel (70 mg/m^2^ per day) and cisplatin (70 mg/m^2^ per day) infusion on day 1, along with 5‐FU (750 mg/m^2^ per day) infusion on days 1–5. Additionally, a single subcutaneous injection of pegfilgrastim at a dose of 3.6 mg was administered on day 3 of each cycle. While antibiotics were not routinely prescribed, physicians had the discretion to administer them if necessary. This treatment cycle was repeated every 3 weeks; for up to 3 cycles or until any of the following occurred: toxicity, patient refusal, or disease progression.

Cisplatin dosage adjustments were implemented if the creatinine clearance (CCr) fell within a specific range: reduced by 20% or 40%, or stopped for a CCr of 50 ≤ CCr < 60, 40 ≤ CCr < 50, or <40 mL/min. Cisplatin was discontinued if grade 2 or higher audiotoxicity occurred. Furthermore, dosage reductions of 20% were applied to 5‐FU, cisplatin, and docetaxel in subsequent cycles in the event of grade 4 neutropenia. Similarly, a 20% reduction was in the dose of 5‐FU and docetaxel was applied in subsequent cycles if grade 3 or 4 stomatitis, esophagitis, or diarrhea occurred.

Following neoadjuvant chemotherapy, patients underwent total or subtotal esophagectomy and regional lymphadenectomy.

### 2.3 Evaluation of adverse events

Adverse events were graded according to CTCAE v 5.0, with blood tests conducted on days 7 and 9 after the first DCF cycle. FN was defined as a neutrophil count less than 1000/μL and a temperature of 38°C or above.

### 2.4 Endpoints

The FN incidence was the primary endpoint. The secondary endpoints included the incidence of grade 3 or higher neutropenia, curative resection rate, preoperative therapy response rate, histopathological tumor response, histopathological complete response rate, adverse event frequency during preoperative therapy, postoperative complication frequency, progression‐free survival (PFS), and overall survival (OS).

Histopathological tumor response was assessed using the JSED histological criteria, categorizing the data into five grades based on the extent of tumor degeneration or necrosis^8^: grade 0, no therapeutic effect; grade 1a, more than 2/3 of the tumor with surviving cancer cells; grade 1b, more than 1/3 of the tumor with surviving cancer cells; grade 2, 1/3 or less of the tumor with surviving cancer cells; and grade 3, no surviving cancer cells in the tumor. RECIST version 1.1 was used to evaluate tumor response in patients with measurable lesions. PFS was defined as the time from the start of preoperative treatment to disease progression or death, while OS was defined as the time from the start of preoperative treatment to death from any cause or the last confirmed survival.

### 2.5 Statistical analysis

We tested the assumption of a null hypothesis indicating an FN incidence of 30%, with an anticipated FN incidence of 10%. With a one‐sided alpha of 0.05 and a desired statistical power of 80%, a minimum sample size of 28 patients was needed, and the projected sample size was set at 35 patients.

To assess the primary endpoint, FN incidence, we calculated the proportion and its 95% confidence interval (CI) using the Clopper–Pearson method. The evaluation involved determining whether the CI overlapped with the predefined threshold of 30%. Adverse events were analyzed by computing frequencies, proportions, and 95% CIs using the Clopper–Pearson method. Additionally, survival analysis was conducted to assess overall survival (OS) and progression‐free survival (PFS) throughout the observation period. The results were visualized through Kaplan–Meier curves. All the statistical analyses were performed using SPSS software (version 28) for Windows (IBM Corporation).

For the univariate analysis of patient characteristics associated with grade 4 neutropenia or febrile neutropenia, the Mann–Whitney U test was used for age, the *t*‐test was used for neutrophil count and creatinine clearance, and Fisher's exact test for sex and prophylactic antibiotics.

## 3 RESULTS

### 3.1 Patient characteristics

**FIGURE 1:**
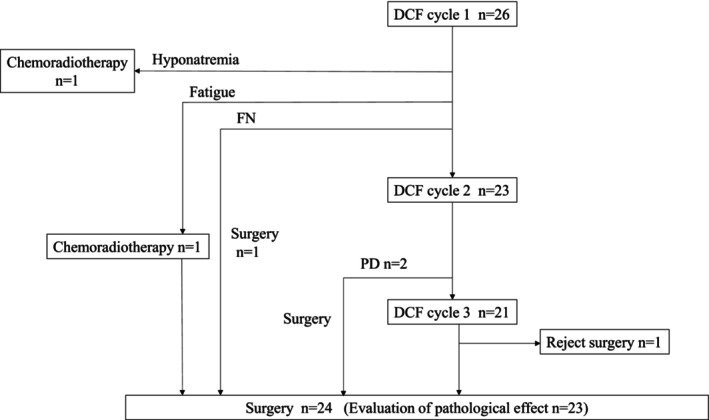
Accrual and treatment summary.

Between March 2020 and January 2022, we enrolled 26 eligible patients in the study, as detailed in Table 1, outlining patient characteristics. The median age of the patients was 66 years, ranging from 41 to 73 years, and all participants exhibited an Eastern Cooperative Oncology Group (ECOG) performance status of 0 or 1. DCF was administered to 26, 23, or 21 patients, via one, two, or three courses, respectively. One patient underwent surgery, one patient received chemoradiotherapy followed by surgery, and one patient received chemoradiotherapy without surgery after one course of DCF. Disease progression occurred in two patients who underwent surgery after two courses of DCF. We administered surgery to 20 patients after three courses of DCF, and one patient refused surgery. Twenty‐four of the 26 patients underwent esophagectomy in total (Figure 1).

**TABLE 1:** Patient characteristics *n* = 26.

Clinical characteristics	No.
Age (years), median (range)	66 (41–73)
Sex	
Male/female	22/4
ECOG performance status	
0/1	24/2
Tumor location	
Ce/Ut/Mt/Lt/Ae	3/4/14/4/1
Histopathological diagnosis	
SCC/Adeno	25/1
Clinical T stage	
1b/2/3	3/5/18
Clinical N stage	
0/1/2/3	0/9/15/2
Clinical M stage	
0/1	22/4
Clinical stage	
II/III/IVA/IVB	3/18/1/4

Abbreviations: Adeno, adenocarcinomaSCC, squamous cell carcinoma.

For the first DCF course, five patients had a creatinine clearance (CCr) between 50 and 60 mL/min, one between 40 and 50 mL/min, and one underwent cisplatin dose adjustments to 80% and 60%, respectively. Dose reduction was necessary for six patients (23.1%). Subsequently, in the second and third courses, dose adjustments were made for 16 out of 23 (69.6%) and 16 out of 21 (76.2%) patients, respectively, due to adverse events from prior courses.

During treatment, one patient experienced chemotherapy delay in the second course due to arthralgia, and another experienced a delay in the third course due to pneumonia. The relative dose intensities for docetaxel, cisplatin, and 5‐FU were 91.6%, 86.6%, and 91.6%, respectively.

Initially, the study commenced with the assumption that FP was the standard of care, and that DCF was an experimental treatment. However, following the publication of JCOG1109 results at the ASCO Gastrointestinal Cancers Symposium in February 2022,^4^ DCF transitioned to the standard treatment subsequently incorporated into the Japanese guidelines.^9^ Consequently, the study halted case accrual, anticipating that DCF would become commonplace in general clinical practice, making future case accumulation challenging due to patients receiving DCF outside clinical trials.

### 3.2 Toxicity of DCF

Table 2 provides an overview of the adverse events attributed to DCF. Among the 26 patients, two (7.7%; 95% confidence interval [CI]: 9.5%–25.1%) had FN in the first course. Notably, grade 3 or higher leukopenia and neutropenia were observed in 13 (50.0%; 95% CI: 29.9%–70.1%) and 15 (57.7%; 95% CI: 36.9%–76.6%) patients, respectively.

**TABLE 2:** Adverse events due to preoperative DCF. *n* = 26.

	Any grade (%)	≥Grade 3 (%)	Grade 3 (%)	Grade 4 (%)
Leukopenia	20 (76.9)	13(50.0)	10 (38.5)	3 (11.5)
Neutropenia	19 (73.1)	15 (57.7)	5 (19.2)	10 (38.5)
Anemia	25 (96.2)	1 (3.8)	1 (3.8)	0 (0)
Thrombocytopenia	24 (92.3)	7 (26.9)	7 (26.9)	0 (0)
Febrile neutropenia	2 (7.7)	2 (7.7)	2 (7.7)	0 (0)
Hyponatremia	22 (84.6)	7 (26.9)	6 (23.1)	1 (3.8)
Anorexia	21 (80.8)	5 (19.2)	5 (19.2)	0 (0)
Hypomagnesemia	18 (69.2)	0 (0)	0 (0)	0 (0)
Increased aspartate aminotransferase	15 (57.7)	0 (0)	0 (0)	0 (0)
Diarrhea	14 (53.8)	6 (23.1)	6 (23.1)	0 (0)
Increased alanine aminotransferase	13 (50)	1 (3.8)	1 (3.8)	0 (0)
Increased creatinine	11 (42.3)	0 (0)	0 (0)	0 (0)
Fatigue	8 (30.8)	1 (3.8)	1 (3.8)	0 (0)
Mucositis	7 (26.9)	2 (7.7)	2 (7.7)	0 (0)
Increased blood bilirubin	7 (26.9)	0 (0)	0 (0)	0 (0)
Nausea	7 (26.9)	0 (0)	0 (0)	0 (0)
Hypokalemia	6 (23.1)	2 (7.7)	1 (3.8)	1 (3.8)
Vomiting	6 (23.1)	0 (0)	0 (0)	0 (0)
Fever	4 (15.4)	0 (0)	0 (0)	0 (0)
Dysgeusia	3 (11.5)	0 (0)	0 (0)	0 (0)
Hiccups	2 (7.7)	0 (0)	0 (0)	0 (0)
Pneumonia	1 (3.8)	1 (3.8)	1 (3.8)	0 (0)
Abdominal pain	1 (3.8)	0 (0)	0 (0)	0 (0)
Arthralgia	1 (3.8)	0 (0)	0 (0)	0 (0)
Tachycardia	1 (3.8)	0 (0)	0 (0)	0 (0)
Stroke	1 (3.8)	0 (0)	0 (0)	0 (0)
Periodontal disease	1 (3.8)	0 (0)	0 (0)	0 (0)

**FIGURE 2:**
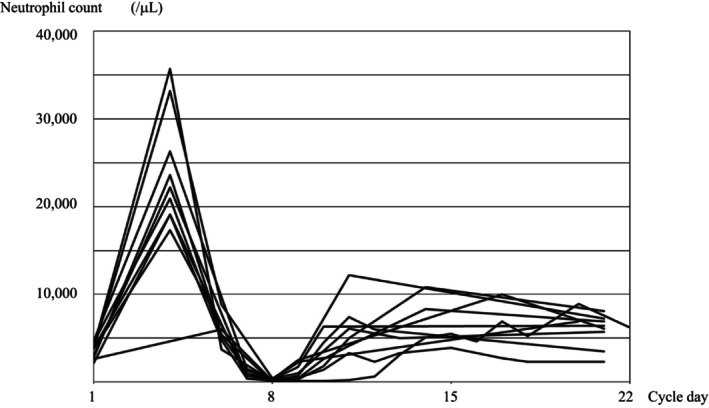
Neutrophil count of patients who experienced febrile neutropenia in the first course of DCF.

Ten out of 26 patients (38.5%; 95% CI: 20.2%–59.4%) had grade 4 neutropenia after the first course of DCF. The physician decided whether to administer prophylactic antibiotics, and we administered oral levofloxacin 500 mg/day on days 5–9 to 12 of the 26 patients. Four of the 14 patients who did not receive prophylactic antibiotics had grade 4 neutropenia, and two of them developed FN. On the contrary, six of the 12 patients who received prophylactic levofloxacin had grade 4 neutropenia, and none had FN. Figure 2 shows the neutrophil counts of patients who experienced grade 4 neutropenia. Other nonhematological adverse events included hyponatremia, anorexia, hypomagnesemia, increased aspartate aminotransferase, and diarrhea (Table 2).

Univariate analysis indicated that age and baseline neutrophil count were associated with grade 4 neutropenia (Table S1). However, due to the limited number of patients with FN, no factors related to FN were identified in the analysis.

### 3.3 Treatment outcome

**FIGURE 3:**
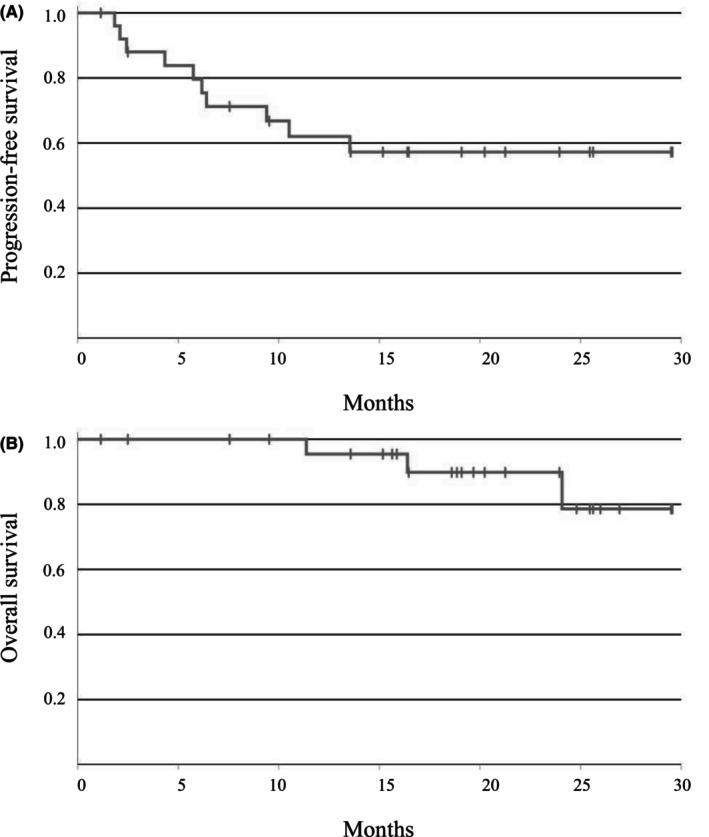
Kaplan–Meier estimates of (A) progression‐free survival and (B) overall survival.

Table 3 illustrates the histopathological response in the subset of 23 patients who underwent surgery following preoperative DCF without additional preoperative treatments, such as radiotherapy. Among these patients, three (11.5%) achieved a histopathological complete response (grade 3), seven (26.9%) had a grade 2 response, five (19.2%) exhibited a grade 1b response, and eight (30.8%) had a grade 1a response. Overall, 10 patients (38.5%) achieved a histopathological response of grade 3 or 2. For the 10 patients with measurable lesions evaluated by RECIST version 1.1, seven (70%) had a partial response (PR), and two (20%) had stable disease (SD) (Table 3). The median follow‐up time for all 26 patients included in the study was 18.6 months, ranging from 1.1 to 29.6 months. The estimated 1‐year PFS was 62.0%, the 1‐year OS was 95.5%, the 2‐year PFS was 57.2%, and the 2‐year OS was 89.8% (Figure 3).

**TABLE 3:** Pathological response and objective response.

Pathological response, *n* = 26		
	No.	(%)
Grade 3	3	(11.5)
Grade 2	7	(26.9)
Grade 1b	5	(19.2)
Grade 1a	8	(30.8)
Not evaluated	3	(11.5)

Objective response, *n* = 10		
	No.	(%)
CR	0	(0.0)
PR	7	(70.0)
SD	2	(20.0)
PD	0	(0.0)
NE	1	(10.0)

Abbreviations: CR, complete response; NE, not evaluable; PD, progressive disease; PR, partial response; SD, stable disease.

Among the 24 patients who underwent esophagectomy (with one patient receiving chemoradiotherapy and 23 patients receiving only DCF as preoperative treatment), Table 4 summarizes the resection margins, including the R0 and R1 resection rates, and postoperative complications.

**TABLE 4:** Summary of resection margins and postoperative complications *n* = 23.

	No. of patients	(%)
Resection margin		
R0	22	(95.7)
R1	1	(4.3)
Postoperative complication		
Anastomotic leakages	3	(13.0)
Recurrent nerve palsy	1	(4.3)
Postoperative mortality	0	(0)

## 4 DISCUSSION

We performed the present study of pegfilgrastim on day 3 to determine if the results of the report by Ishikawa et al. could be reproduced in which Grade 4 neutropenia occurred in two of 23 patients (8.7%) and FN in 0%.^7^ However, in this study, 10 of 26 patients (38.5%) had grade 4 neutropenia, and two of 26 patients (7.7%) had FN after the first course of DCF, even though we used pegfilgrastim on day 3 as a prophylactic measure. In the present study, antibiotics were not mandatory and were not used at the discretion of the physician in charge initially because we expected the frequency of Grade 4 neutropenia to be very low. However, both Grade 4 neutropenia and FN occurred in significant numbers, so antibiotics were used beginning in the middle of patient enrollment at the discretion of the physician. Four of the 14 patients who did not receive antimicrobial prophylaxis had grade 4 neutropenia, and two of them had FN. On the contrary, none of the 12 patients who received levofloxacin prophylaxis had FN, even though six of them had grade 4 neutropenia.

In a previous study in which pegfilgrastim was administered on day 7, the nadir of neutropenia was on day 9,^6^ so it was expected to reach its nadir in the vicinity. Although the protocol required blood tests on day 7 and day 9, blood tests were also performed on other days. Blood tests were performed on day 8 for 18 of the 26 patients with expected neutropenia on day 7, and the possibility that grade 4 neutropenia was missed in the remaining 10 patients could be completely ruled out.

The prescribing information for pegfilgrastim recommends administration at least 24 h after myelosuppressive chemotherapy completion because G‐CSF causes myeloid progenitor cells to divide rapidly, and these cells are especially sensitive to cytotoxic chemotherapy.^5^ It is recommended that pegfilgrastim be administered 24–72 h after chemotherapy according to the clinical practice guidelines of the American Society of Clinical Oncology (ASCO),^10^ the National Comprehensive Cancer Network (NCCN),^11^ and the European Organization for Research and Treatment of Cancer (EORTC),^12^ because the administration of G‐CSF at earlier times may increase myelosuppression. Therefore, in most studies, pegfilgrastim is administered on day 7, the day after the continuous infusion of 5‐FU ends.

Retrospective studies indicated that pegfilgrastim prophylaxis on day 7 reduced the risk of FN, but the incidence of FN varied (3–30%) (Table 5).^13^, ^14^, ^15^, ^16^ Our previous prospective study of DCF combined with pegfilgrastim on day 7 reported a 29.7% incidence of FN.^6^ On the contrary, the results of a prospective study showed that DCF combined with pegfilgrastim on day 3 resulted in a low grade 4 neutropenia rate of 8.7% (95% confidence interval: 4.7–22.1) in two of 23 patients and an FN rate of 0% with prophylactic use of levofloxacin on days 5–9 (Table 5).^7^

**TABLE 5:** Previous reports of preoperative DCF with prophylactic use of pegfilgrastim.

	Author	Ref	Year	Type of study	Docetaxel	Cisplatin	Fluorouracil
					(mg/m^2^)	(mg/m^2^)	(mg/m^2^)
1	Yoshida	17	2018	Retrospective	70	70	700 × 5
2	Kawahira	18	2018	Retrospective	70	70	750 × 5
3	Ohkura	19	2019	Retrospective	75	75	750 × 5
4	Okamoto	20	2022	Retrospective	60–70	60–70	750 − 800 × 5
5	Ishikawa	11	2019	Prospective	70	70	750 × 5
6	Maeda	10	2022	Prospective	70	70	750 × 5
7	Present study			Prospective	70	70	750 × 5

	Administration of G‐CSF	Antibiotics	Number of	G4 neutropenia	Febrile neutropenia		
			patients	*n*	(%)	*n*	(%)
1	Pegfilgrastim on day 7 or later	(not described)	35^a^	4	11.4	4	11.4
2	Filgrastim or pegfilgrastim on day 7	CPFX or LVFX days 5–15	26	11	42.3	8	30.8
3	Pegfilgrastim on day 7	None	33	–	–	1	3.0
4	Pegfilgrastim on day 7	(not described)	65	10	15.4	8	12.3
5	Pegfilgrastim on day 3	LVFX on days 5–9	23	2	8.7	0	0
6	Pegfilgrastim on day 7	None	37	11	29.7	11	29.7
7	Pegfilgrastim on day 3		26	10	38.5	2	7.7
		None	14	4	28.6	2	14.3
		LVFX on days 5–9	12	6	50	0	0

^a^ Number of administrations.

Abbreviations: CPFX, ciprofloxacin; LVFX, levofloxacin.

We were unable to determine the reason for the difference in the incidence of FN among the studies. Ohkura et al. reported that patient age is a risk factor for FN.^15^ The median age of the patients in Ishikawa et al.'s study was 62 years (range: 44–75), and that of patients in this study was 66 years (41–73).^7^ Our patients were slightly older, but we do not know if this caused the difference in the FN rate.^6^

According to the definition of FN, in CTCAE v 5.0, FN is defined as an “absolute neutrophil count <1000/mm^3^ with a single temperature of >38.3°C or a sustained temperature of ≥38°C for more than one hour”. However, in the present study, we used the same FN criteria as those used in a report by Ishikawa et al.,^7^ that is, a neutrophil count less than 1000/μL and a temperature of 38°C or above. After reviewing the clinical course in our study, the same patients were diagnosed with FN at the same time using either criterion. For the sake of generalization, it would have been better to use the definition of CTCAE.

Prophylactic treatment with levofloxacin has been reported to prevent FN in patients with neutropenia from cancer chemotherapy.^17^ However, ASCO clinical practice guidelines recommend prophylactic antibiotics only when neutropenia less than 100/μL lasts longer than 7 days.^18^ According to a report of DCF therapy combined with prophylactic antimicrobial agents and without prophylactic G‐CSF administration, grade 4 neutropenia occurred in 45.2% of patients, and febrile neutropenia occurred in 2.4%.^19^ The clinical need for prophylactic antibiotics for patients receiving DCF should be carefully considered in considering the risk of emergence of resistant bacterial strains.

This study has several limitations. First, this study included a small number of patients treated with or without prophylactic antibiotics; therefore, a larger study, preferably a randomized study, is needed to test whether the prophylactic use of pegfilgrastim and antimicrobial agents can reliably prevent FN. Second, since we allowed prophylactic antibiotics based on the physicians' judgment, the FN rate may have been affected even though the main goal was to determine the FN rate. Third, since we reduced the cisplatin dose in six of the 26 patients in the first cycle because of kidney problems, we cannot rule out the possibility that we underestimated the FN rate by reducing the cisplatin dose. Fourth, the study included 28 patients, but enrollment was terminated after 26 patients were enrolled. If the remaining two patients developed FN, the primary endpoint was not met.

In summary, DCF is effective as a preoperative chemotherapy regimen, but pegfilgrastim administered on day 3 may not be enough to prevent FN, and the use of both antibiotics and pegfilgrastim may be promising options. A larger number of patients should be tested in clinical trials using both prophylactic G‐CSF and prophylactic antibiotics to confirm the efficacy of prophylaxis for FN.

## AUTHOR CONTRIBUTIONS

**Osamu Maeda:** Conceptualization (equal); investigation (equal); writing – original draft (lead). **Satoshi Furune:** Investigation (equal); writing – review and editing (equal). **Mitsuro Kanda:** Investigation (equal); writing – review and editing (equal). **Kazushi Miyata:** Investigation (equal); writing – review and editing (equal). **Dai Shimizu:** Investigation (equal); writing – review and editing (equal). **Shizuki Sugita:** Investigation (equal); writing – review and editing (equal). **Kazuki Nishida:** Data curation (equal); investigation (equal); writing – original draft (equal). **Masahiko Ando:** Data curation (equal); writing – review and editing (equal). **Yasuhiro Kodera:** Conceptualization (equal); supervision (equal); writing – review and editing (equal). **Yuichi Ando:** Conceptualization (equal); supervision (lead); writing – review and editing (equal).

## CONFLICT OF INTEREST STATEMENT

Yasuhiro Kodera reports personal fees from Ono Pharmaceutical Co., Ltd. and Daiichi Sankyo Co., Ltd. and research funds from Chugai Pharmaceutical Co., Ltd., Taiho Pharmaceutical Co., Ltd., and Yakult Honsha Co., Ltd. Yuichi Ando reports personal fees from Chugai Pharmaceutical Co., Ltd., personal fees from Bayer Holding Ltd., research funds from Beigene, and grants from Geo Holdings related to efforts other than the submitted work. The other authors have no conflicts of interest to declare.

## ETHICS STATEMENT

The study was designed and conducted in line with the Helsinki Declaration and the Ethical Guidelines for Clinical Research (Ministry of Health, Labor and Welfare, Japan). Approval of the research protocol by an institutional review board: This study was approved by the Institutional Review Board (approval no. 2019–0518).

## INFORMED CONSENT

All patients provided written informed consent.

## Supporting information


Table S1

## Data Availability

The datasets used and/or analyzed during the current study are available from the corresponding author upon reasonable request.
