# Supplementary material for: Docetaxel, cisplatin, and fluorouracil with pegfilgrastim on day 3 as neoadjuvant chemotherapy for esophageal cancer
Source: Cancer Med. 2024 Feb 1;13(2):e6974. doi: 10.1002/cam4.6974 (PMC10831909; doi:10.1002/cam4.6974)
Supplement: Supplementary file 1 — Table S1 [file CAM4-13-e6974-s001.docx]

**TABLE S1.** Characteristics of patients with or without grade 4 neutropenia and febrile neutropenia

|  | Grade 4 neutropenia | |  |
| --- | --- | --- | --- |
|  | yes (n=10) | no (n=16) | p value |
| Age (median, range) | 71, 52-73 | 64, 41-72 | 0.004 |
| Sex |  |  | 0.625 |
| male | 8 | 14 |  |
| female | 2 | 2 |  |
| Neutrophil count (mean ±SD) | 3620 ± 989 | 4763 ± 1513 | 0.029 |
| Creatinine clearance (mean ±SD) | 68.5 ± 12.6 | 80.2 ± 21.6 | 0.094 |
| Prophylactic antibiotics |  |  | 0.422 |
| yes | 6 | 6 |  |
| no | 4 | 10 |  |
|  |  |  |  |
|  |  |  |  |
|  | Febrile neutropenia | |  |
|  | yes (n=2) | no (n=24) | p value |
| Age (median, range) | 69.5, 69-70 | 65, 41-73 | 0.394 |
| Sex |  |  | 1 |
| male | 2 | 20 |  |
| female | 0 | 4 |  |
| Neutrophil count (mean ±SD) | 3350 ± 919 | 4404 ± 1149 | 0.327 |
| Creatinine clearance (mean ±SD) | 76.2 ± 9.0 | 75.7 ± 20.0 | 0.975 |
| Prophylactic antibiotics |  |  | 0.483 |
| yes | 0 | 12 |  |
| no | 2 | 12 |  |
